# Characterizing Peri-Implant and Sub-Gingival Microbiota through Culturomics. First Isolation of Some Species in the Oral Cavity. A Pilot Study

**DOI:** 10.3390/pathogens9050365

**Published:** 2020-05-10

**Authors:** Leonardo Martellacci, Gianluca Quaranta, Giovanni Fancello, Antonio D’Addona, Maurizio Sanguinetti, Romeo Patini, Luca Masucci

**Affiliations:** 1 Private Practice, 00168 Rome, Italy; lmartellacci@virgilio.it; 2 Department of Laboratory and Infectious Sciences, Fondazione Policlinico Universitario A. Gemelli IRCCS, Università Cattolica del Sacro Cuore, 00168 Rome, Italy; gquaranta88@gmail.com (G.Q.); maurizio.sanguinetti@unicatt.it (M.S.); luca.masucci@unicatt.it (L.M.); 3 Department of Head, Neck and Sense Organs, Division of Oral Surgery and Implantology, Fondazione Policlinico Universitario A. Gemelli IRCCS, Università Cattolica del Sacro Cuore, 00168 Rome, Italy; antonio.daddona@policlinicogemelli.it; 4 Department of Basic Biotechnological Sciences, Intensive and Perioperative Clinics, Università Cattolica del Sacro Cuore, 00168 Rome, Italy; 5 Department of Head, Neck and Sense Organs, Division of General Dentistry and Orthodontics, Fondazione Policlinico Universitario A. Gemelli IRCCS, Università Cattolica del Sacro Cuore, School of Dentistry, 00168 Rome, Italy; romeo.patini@unicatt.it

**Keywords:** culturomics, oral microbiota, implant dentistry, periodontology, pilot study

## Abstract

Background: In recent years, culture-independent molecular techniques have been developed to investigate microbiota considered uncultivable. However, the data in the literature suggest that molecular techniques and cultural methods target different spectra of bacteria. The objective of this pilot study was to search for not yet identified oral species in the peri-implant and sub-gingival microbiota in patients without signs of oral pathologies, through the use of the culturomics approach, which has never been used before in dentistry. Methods: Four patients were enrolled; from each patient, samples of sub-gingival and peri-implant plaque were taken and analysed by culturomics. Results: Of 48 isolated species, only 30 had been previously identified by metagenomics in other studies; on the contrary, 12 species had never been associated with the oral cavity before, and 5 of them had never been isolated from clinical specimens. Conclusions: By adopting culturomics in dentistry, it could be possible to identify a large amount of fastidious microorganisms that inhabit the oral cavity and to more accurately characterize the microorganisms that lead to periodontitis and peri-implantitis. This evidence could represent an important step forward for the diagnosis and treatment of peri-implantitis, as well as a very useful means for the characterization of new potential aetiologic agents.

## 1. Introduction

It was estimated that more than 700 microbial species could inhabit the oral cavity and that each individual could host up to 150 microbial species. Even in sub-gingival sites, the amount of bacteria ranges from 10^3^ to 10^8^ bacteria [1], and only a few microorganisms play a prominent role in the establishment of pathological processes affecting periodontal and peri-implant tissues [2]. In the study of such periodontal pathogens, the major difficulties encountered by microbiologists have been technical limits, sample recruitment, dispersion of plaque during sampling, cultivation and isolation of bacterial species, oral microbiota complexity, and data analysis [3]. These limits have been partly bypassed using several approaches [4]: immunoblot techniques to study some selected species [5,6], checkerboard DNA–DNA hybridization techniques [7,8], and DNA probes [9]. In the most recent years, new technologies, such as metagenomics, have been developed to investigate the fastidious bacteria that inhabit the human microbiota [10]. The results obtained with this technique highlighted that many more species than previously presumed could be found in the oral cavity [11,12]. However, some studies have suggested that molecular techniques and cultural methods target a different spectrum of bacteria [10]. Therefore, in the recent years, there has been a rebirth of culture methods. This revisited approach has been defined as culturomics. It consists of the use of different culture conditions, such as selective liquid and/or solid media, variable temperature, and time of incubation [13,14].

Metagenomics and culturomics are different analytical methods that pursue the same objective: the detection of as many microbial species in a particular ecosystem as possible. Several strengths can be attributed to metagenomics: the possibility of analysing a high number of samples (up to 96 samples), the speed of analysis results (3–5 days with statistical analysis), and the possibility of quantifying the single clusters of microorganisms in terms of percentage (relative abundance) [14]. Some weaknesses of the metagenomic approach must also be noted: the efficacy of DNA extraction is a crucial operator-dependent step that could affect the repeatability of the test [13]. Moreover, this method cannot discriminate between live bacteria and transient DNA and produces a large amount of output data, which need to be analysed by a statistician. Finally, depth bias is probably considered the major limitation of metagenomics. In fact, bacterial species with concentrations lower than 10^5^ cells per gram cannot be detected with metagenomic analysis, thus the minority populations are not correctly or partly detected [14]. This aspect affects genomic analyses in dentistry because plaque samples are very rich in species, but often poor in quantity. On the other hand, culturomics techniques allow the cultivation and identification of a large number of bacterial species that are hardly detectable by metagenomics [10]. This method is able to detect bacteria regardless of their relative abundance and can easily recognize only living microorganisms; other microbiological parameters, such as virulence factors, sensitivity, or resistance to antibiotics, can also be studied with this method. Moreover, species identification by mass spectrometry (MALDI-TOF) is very fast (1 min for each colony) and accurate. The major limits of this approach are that it is technically laborious and it takes a long time to obtain the results [14].

The purpose of this research project was to investigate the peri-implant and sub-gingival microbiota in patients rehabilitated with dental implants (6 mm micro-rough short implants, Straumann and tissue-level standard implants, Straumann) in the absence of any type of peri-implant or periodontal disease with the use of a culturomics, which has not yet been used in dentistry.

## 2. Results

The baseline characteristics of the enrolled patients are reported in Table 1.

**Figure 1:**
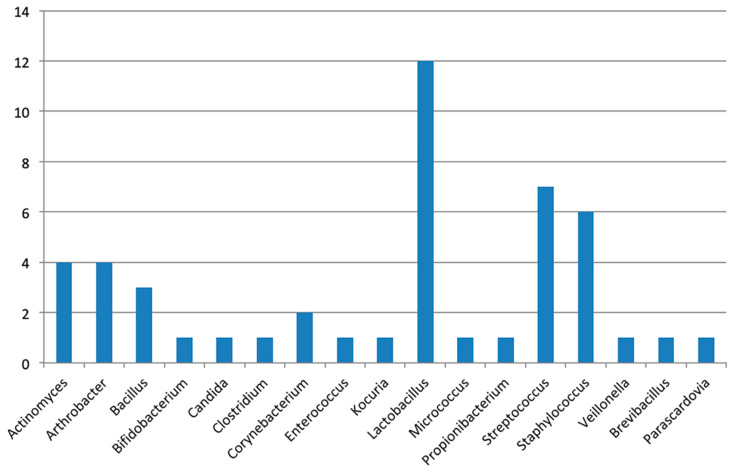
Species identified in the patients enrolled in the study, distributed among their 17 genera. On the *y*-axis, the number of species identified is reported; on the *x*-axis, the genera isolated in this study are reported.

From the culturomics analysis of the peri-implant and sub-gingival plaque of the 4 patients enrolled in this study, 48 microbial species were identified. Such species belong to 17 different genera (Figure 1).

Isolated colonies were identified by MALDI-TOF mass spectrometry with scores over 1.8. All the identified species are reported in Table 2.

## 3. Discussion

The present study represents the first application of the culturomics approach for the analysis of the periodontal and peri-implant microbiota in dentistry, focusing on patients with short dental implants with clinically healthy periodontal and peri-implant tissues. The analysis of the oral samples led to the identification and cultivation of bacterial species never before isolated from the oral cavity. Such species included *Arthrobacter gandavensis*, *Arthrobacter oxydans*, *Arthrobacter protophormaie*, *Arthrobacter tumbae*, *Bacillus bataviensis*, *Bacillus licheniformis*, *Bacillus luciferensis*, *Brevibacillus borstelensis*, *Clostridium novyi*, *Corynebacterium flavescens*, *Corynebacterium lipophiloflavum*, and *Kocuria marina.* Moreover, some of these species were never previously associated with human microbiota: *A*. *gandavensis*, *A*. *tumbae*, *B*. *bataviensis*, *B*. *luciferensis*, and *B*. *borstelensis.* Concerning patient 1, 14 species were detected in the peri-implant sulcus and 14 in the sub-gingival plaque, for a total of 22 different species. In both samples examined, whether they were incubated for 7 or 14 days, a similar distribution of species was noted [15,16,17,18,19]. *A*. *gandavensis* was isolated from a short implant sample of the same patient. This bacterium was previously isolated from mammary and uterine infections in cattle, and its pathogenic role in these processes is still uncertain [20]. The pathogenic species and the opportunistic pathogens identified from patient 1 were *Bifidobacterium dentium*, *Candida albicans*, *C*. *novyi*, *Enterococcus faecalis*, *Micrococcus luteus*, and *Propionibacterium acnes*. Among them, the finding of *Clostridium novyi* aroused particular interest, as it had never been isolated from dental plaque before. This species has been demonstrated to cause clostridial myonecrosis in animals and humans [21] as well as severe septicaemia [22]. From the sample of patient 2, *C*. *flavescens* and *C*. *lipophiloflavum* were isolated; these bacteria are considered opportunistic pathogens of other body districts: the former causes superficial infection of the skin [23], and the latter causes vaginitis [24]. Another species isolated from patient 2 was *Kocuria marina*. This bacterium is currently the focus of numerous studies, as it appears to be a pathogen affecting mainly dialysis patients [25,26]. One of the most interesting species isolated from patient 2 is *B*. *licheniformis*. It is capable of synthesizing a nitrogen-rich exopolysaccharide, which has important anti-tumor and tissue healing promotion properties [27]. This bacterium also produces the enzyme NucB, which inhibits biofilm formation on hard tissues of the oral cavity [28]. Another microorganism isolated from patient 2 is *B*. *borstelensis*. This peculiar bacterium is able to degrade plastics and some other inorganic compounds [29]. Eleven species were isolated from patient 3, and 15 species were isolated from patient 4. Among them, some species not yet associated with the oral cavity were found: *A*. *tumbae*, *B*. *bataviensis*, *L*. *buchneri*, and *L*. *parabuchneri*. *Arthrobacter tumbae*, like the other members of the genus *Arthrobacter*, has not yet been associated with the oral cavity, thus this strain could be considered the first strain cultivated and isolated from human oral cavity specimens. The culturomics analysis also revealed two members of the Bacillus genus that had never before been isolated from humans: *B*. *bataviensis*, considered as an environmental microorganism [30], and *B*. *luciferensis* from the sub-gingival plaque sample. It was demonstrated that the latter is able to obtain the pX02 gene from *Bacillus anthracis*; this gene encodes a protein involved in the formation of the bacterial capsule, which is the major virulence factor of *Bacillus anthracis* [31]. Some limitations of the study must also be highlighted. The study design and the paucity of patients enrolled probably represent the major study limitation. In fact, even if culturomics allowed the deep and accurate descriptive analysis of the oral microbiota, the small number of samples precluded the possibility of identifying any microorganism–disease correlation. Furthermore, as demonstrated by the results of this study, culturomics represents a suitable method to personalize the diagnosis and therapy of oral diseases. Since this study is the first to apply culturomics in dentistry, this paper can also be considered as a demonstration of how to apply the method. Metagenomics and culturomics should be considered as complementary strategies to identify new potential pathogens and then to prompt focused antibiotic therapies [32,33]. This pilot study, along with similar articles about the association between periodontal and systemic diseases [34,35,36,37,38,39], provided some new information about the complexity of the tooth and peri-implant microbiota. Future perspectives will be to expand the studied cohorts to add predictive value to the detected species and to establish a medium–long follow-up to accurately study plaque composition over time.

## 4. Materials and Methods

### 4.1. Patient Recruitment and Sample Collection

In the period between November 2018 and January 2019, in a private practice dental office, four patients corresponding to the inclusion and exclusion criteria were consecutively enrolled. The inclusion criteria were as follows: patient rehabilitated with short dental implants (Standard Plus Tissue Level Straumann implants 4.8 mm diameter and 6 mm length, SLA surface) bearing prostheses for at least 1 year, no systemic disease that could modify the immune response, non-smokers, absence of active oral disease, and absence of antibiotic therapy in the 3 months before sampling. As the study is a pilot study for the analysis of dental or peri-implant plaque with culturomics, the calculation of the sample size was not applicable. This pilot study was conducted according to the 1975 Helsinki Declaration, as revised in 2000. The study was approved by the local ethical committee, with approval number 0042686/18. Informed consent was obtained from each patient. The area selected for sampling was the vestibular surface of molars, since, as demonstrated by Teles et al., the average bacterial count is greatest in these sites [2]. Sampling from vestibular surfaces also avoids contamination from contact with the mucous surface. The sub-gingival plaque samples were taken after isolating the area from saliva and removed by an aspirator for approximately 30 s. The plaque from the implant and tooth surfaces in the most apical area of the peri-implant or periodontal sulcus was sampled using a sterile implant deplaquer (Hawe-neos) and a sterile steel curette (SGR 11/12 and SGR 13/14, Hu-Friedy), respectively. The samples obtained were immediately placed in the primary container (ESwab, COPAN) and transported to the microbiology laboratory at room temperature within 45 min.

### 4.2. Culturomics Protocol

**Figure 2:**
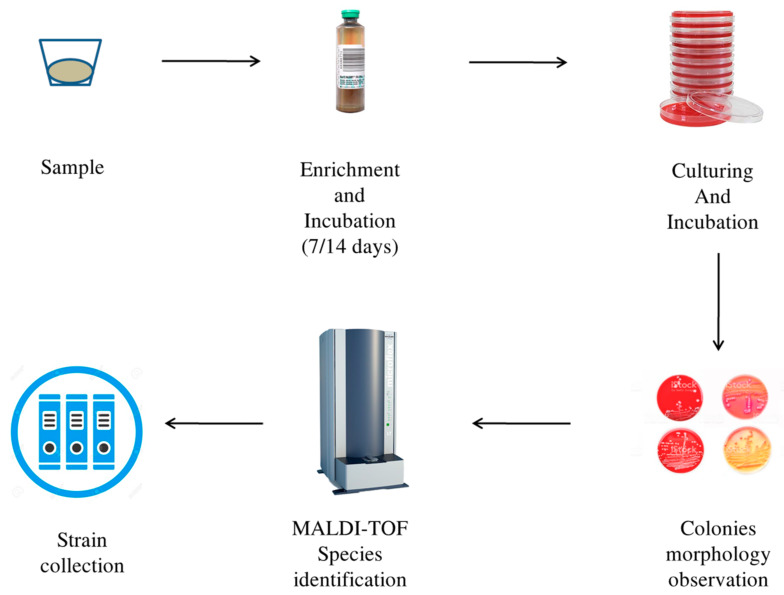
Culturomics flowchart.

As soon as it arrived in the laboratory, the sample was inoculated and incubated in 3 mL of Brucella Broth (Benkton-Dickinson, Franklin Lakes, NJ, USA) and centrifuged (10 min, 3000× *g* rpm). The obtained pellet was resuspended in 6 mL of Thyoglicollate Broth (THIO) (Thermo Fisher Scientific, Waltham, MA, USA) and vortexed. The suspension was equally divided into two sterile vials. A total of 2 mL of Brucella Broth or 2 mL of THIO was added to these two vials. The content of the vial with THIO and sample was inoculated into six pairs of blood culture bottles: 2.5 mL in an aerobic bottle and 2.5 mL in an anaerobic bottle. Each pair of bottles was incubated with stirring for 7 days and 14 days at 37 °C, 30 °C, and 42 °C. After the incubation period (7 days and 14 days), the sample was inoculated on agar culture media. The growth conditions used were as follows: TSA agar (Trypticase soy agar + 5% sheep blood, BioMérieux, Craponne, France), CNA (Columbia agar + 5% blood of sheep, BioMérieux, Craponne, France) for aerobes, and PVX (PolyViteX chocolate agar, BioMérieux, Craponne, France) for fastidious bacteria. The plates were incubated at 37 °C, 30 °C, and 42 °C under aerophilic and micro-aerophilic conditions, respectively (Table 3). The contents of the anaerobic blood culture bottle were inoculated on six plates: three were incubated in micro-aerophilia, and three were incubated in an anaerobic environment. The media chosen for microaerophilia and anaerobiosis were the same for the two conditions: Schaedler agar with kanamycin–vancomycin agar with 5% sheep blood (BioMérieux, Craponne, France), CNA agar, and PVX agar. To recreate the micro-aerophilic conditions, the media were incubated in an oxygen-impermeable plastic bag, inside which a Campygen compact (Oxoid, Lenexa, KS, USA) sachet was inserted. Anaerobic conditions were obtained using an anaerobic hood. The plates were incubated for five days at 37 °C. After five day, any colony found on growth media was sub-cultivated using the same culture media and the same growth conditions described above. Subsequently, species identification was performed by MALDI-TOF mass spectrometry (Bruker-Daltonics, Billerica, MA, USA). This instrument allows rapid identification of examined colonies. For this method, each pure colony was spotted on a metallic plate and then overlaid with 1 μL of alpha-cyano-4-hydroxycinnamic acid. When the plate was in position, a laser beam was positioned on the colonies spotted on the plate. In this way, the proteins of the microorganisms became volatile and moved in relation to their charge and mass. The result was a spectrum profile [15]. Each bacterial profile was compared with a software database (version 4.1.60) (Bruker-Daltonics, Billerica, MA, USA). The spectral homology was converted into a score ranging from 1.2 to values greater than 2.0. Scores above 1.8 denote a high probability of identification success. The results were compared with the morphological aspect of the colonies (α, β, and γ haemolysis, diameter, smell, pigmentation, smoothness, or roughness). The culturomics flowchart is described in Figure 2. Any uncertain result provided by MALDI (score below 1.7) was verified by 16S gene sequencing analysis of the colonies [13,14].

### 4.3. 16S rRNA Gene Sequencing

DNA extraction was performed using the QIAamp Fast DNA Mini Kit (Qiagen, Hilden, Germany). The DNA was quantitated by a Thermo Fisher Scientific NanoDrop ND-1000 spectrophotometer. A negative control (molecular biology-grade pure water) was extracted and then processed together with the other samples. The V3-V4 regions of the 16S rRNA gene were amplified using degenerate forward (Pro341F: 5′-CCTACGGGNBGCASCAG-3′) and reverse (Pro805R: 5′-GACTACNVGGGTATCTAATCC-3′) primers. The sequences obtained were compared in terms of homology with online databases (https://blast.ncbi.nlm.nih.gov/Blast.cgi).

## 5. Conclusions

This pilot study was designed to demonstrate that culturomics could be used to obtain information for the early diagnosis and personalized therapy of peri-implantitis. Even with the paucity of enrolled patients, the data collected in this study are encouraging. In fact, of 48 isolated species, only 30 had been previously identified by metagenomics in clinical studies; 12 species were never previously associated with the oral cavity, and 5 of them were never isolated from clinical samples. These data demonstrate that culturomics is an effective technique to study microorganisms inhabiting the oral cavity, even fastidious microorganisms, once considered “uncultivable”. For a better understanding of the composition of the sub-gingival and peri-implant microbiota, it will be necessary to design larger studies comparing different categories of patients and with a longer follow-up in the future.

From the abovementioned evidence, culturomics could be considered as an easy protocol and a tool to personalize medical treatments. Future studies should associate the clinical status of a patient with the microbiological results obtained with culturomics and perhaps with metagenomics.

## Figures and Tables

**Table 1:** Baseline characteristics of the patients enrolled in the study. The sampling sites for both dental implants and teeth are reported for each subject.

Patient (Sex)	Age	Smoke	Antibiotic Therapy	Systemic Disease	Active Oral Disease	Implant Site	Implant PD/BoP	Tooth Site	Tooth PD/BoP	Prosthesis
1 (F)	36	No	None	None	None	3.7	2/Absent	3.6	3/None	Crown
2 (F)	62	No	None	HP	None	4.6	2/Absent	NA	NA	Toronto Bridge
3 (F)	73	No	None	None	None	4.5	1/Absent	4.4	1/None	Crown
4 (F)	68	No	None	HP	None	3.6	2/Absent	3.5	2/None	Crown

PD, probing depth; BoP, Bleeding on Probing; HP, Hypertension; NA, Not available.

**Table 2:** Summary of the species isolated from each patient and each sampling site after culture enrichment.

Patient	Samples Site	Culture Time	Species Identified by Culturomics
P 1	implant	7 days	*A*. *odontolyticus*, *A*. gandavensis *, *B*. *dentium*, *L*. *casei*, *L*. *fermentum*, *L*. *gasseri*, *L*. *rhamnosus*, *S*. *capitis*, *S*. *gordonii*, *S*. *haemolyticus*
		14 days	*L*. *fermentum*, *L*. *gasseri*, *L*. *rhamnosus*, *M*. *luteus*, *P*. *acnes*, *S*. *epidermidis*, *S*. *mutans*
	tooth	7 days	*A*. *odontolyticus*, *A*. *oris*, *C*. *albicans*, *C*. novyi **, *E*. *faecalis*, *L*. *fermentum*, *L*. *gasseri*, *L*. *salivarius*, *S*. *epidermidis*, *S*. *capitis*, *S*. *cohnii,*
		14 days	*A*. *odontolyticus*, *A*. *oris*, *C*. *albicans*, *L*. *fermentum*, *L*. *kitasatonis*, *L*. *salivarius*, *S.simulans*
P 2	implant	7 days	*A*. *odontolyticus*, *B*. *dentium*, *C*. flavescens **, *K*. marina **, *L*. *reuteri*, *S*. *epidermidis*, *S*. *gordonii*
		14 days	*A*. *odontolyticus*, *A*. *oris*, *A*. oxydans **, *A*. protophormiae **, *B*. licheniformis **, *B*. *dentium*, *B*. borstelensis *, *C*. lipophiloflavum **, *L*. *plantarum*, *L*. *reuteri*, *M*. *luteus*, *P*. *denticolens*, *P*. *acnes*, *S*. *capitis*, *S*. *epidermidis*, *S*. *haemolyticus*
P 3	implant	7 days	*B*. *dentium*, *S*. *epidermidis*, *S*. *hominis*, *S*. *intermedius*, *S*. *mutans*, *S*. *salivarius*
		14 days	*A*. *naeslundii*, *B*. *dentium*, *L*. *gasseri*, *S*. *epidermidis*, *S*. *hominis*, *S*. *mutans*
	tooth	7 days	*A*. *oris*, *S*. *mitis*
		14 days	*P*. *acnes*, *S*. *epidermidis*, *S*. *agalactiae*
P 4	implant	7 days	*A*. *odontolyticus*, *B*. *dentium*, *C*. *albicans*, *E*. *faecalis*, *L*. *delbrueckii*, *L*. *fermentum*, *L*. *paracasei*, *L*. *rhamnosus*, *M*. *luteus*, *S*. *epidermidis*, *V*. *parvula*
		14 days	*A*. *dentalis*, *A*. *odontolyticus*, *A*. tumbae*, *B*. bataviensis *, *B*. *dentium*, *C*. *albicans*, *E*. *faecalis*, *L*. *casei*, *L*. *fermentum*, *L*. *gasseri*, *L*. *paracasei*, *L*. *rhamnosus*
	tooth	7 days	*A*. *odontolyticus*, *B*. luciferensis *, *C*. *albicans*, *E*. *faecalis*, *L*. *gasseri*, *L*. *rhamnosus*, *S*. *capitis*
		14 days	*E*. *faecalis*, *L*. *gasseri*, *L*. *rhamnosus*, *L*. *buchneri*, *L*. *parabuchneri*

P, patient; the days refer to incubation time (see Materials and Methods); in brackets, sampling site; * never associated with humans before; ** first isolation from oral sample.

**Table 3:** Media and culture conditions used in this study.

Pre-incubation in aerobic blood culture bottle for 7 and 14 days at 30 °C. Culturing on TSA, CNA, PVX in aerobic condition at 30 °C.
Pre-incubation in aerobic blood culture bottle for 7 and 14 days at 30 °C. Culturing on TSA, CNA, PVX in microaerophilic condition at 30 °C.
Pre-incubation in anaerobic blood culture bottle for 7 and 14 days at 30 °C. Culturing on CNA and SCH in anaerobic condition at 30 °C.
Pre-incubation in aerobic blood culture bottle for 7 and 14 days at 37 °C. Culturing on TSA, CNA, PVX in aerobic condition at 37 °C.
Pre-incubation in aerobic blood culture bottle for 7 and 14 days at 37 °C. Culturing on TSA, CNA, PVX in microaerophilic condition at 37 °C.
Pre-incubation in anaerobic blood culture bottle for 7 and 14 days at 37 °C. Culturing on CNA and SCH in anaerobic condition at 37 °C.
Pre-incubation in aerobic blood culture bottle for 7 and 14 days at 42 °C. Culturing on TSA, CNA, PVX in aerobic condition at 42 °C.
Pre-incubation in aerobic blood culture bottle for 7 and 14 days at 42 °C. Culturing on TSA, CNA, PVX in microaerophilic condition at 42 °C.
Pre-incubation in anaerobic blood culture bottle for 7 and 14 days at 42 °C. Culturing on CNA and SCH in anaerobic condition at 42 °C.
